# Computational and *in vitro* evaluation of sumac-derived ^©^Rutan compounds towards Sars-CoV-2 M^pro^ inhibition

**DOI:** 10.3389/fphar.2025.1518463

**Published:** 2025-02-04

**Authors:** Muzaffar Kayumov, Parthiban Marimuthu, Jamoliddin Razzokov, Nurkhodja Mukhamedov, Akmal Asrorov, Nodir S. Berdiev, Jamolitdin F. Ziyavitdinov, Ansor Yashinov, Yuliya Oshchepkova, Shavkat Salikhov, Sharafitdin Mirzaakhmedov

**Affiliations:** ^1^ Institute of Bioorganic Chemistry, AS of Uzbekistan, Tashkent, Uzbekistan; ^2^ Pharmaceutical Science Laboratory (PSL-Pharmacy), Structural Bioinformatics Laboratory (SBL-Biochemistry), Faculty of Science and Engineering, Åbo Akademi University, Turku, Finland; ^3^ Center for Global Health Research, Saveetha Medical College, Saveetha Institute of Medical and Technical Sciences, Chennai, India; ^4^ Institute of Fundamental and Applied Research, National Research University TIIAME, Tashkent, Uzbekistan; ^5^ Department of Natural Sciences, Shakhrisabz State Pedagogical Institute, Shahrisabz, Uzbekistan; ^6^ Department of Biotechnology, Tashkent State Technical University, Tashkent, Uzbekistan; ^7^ Department of Natural Compounds and Applied Chemistry, National University of Uzbekistan, Tashkent, Uzbekistan; ^8^ Shanghai Institute of Materia Medica, Chinese Academy of Sciences, Shanghai, China

**Keywords:** Rutan, SARS-CoV-2 M^pro^, docking, MD simulations, *in vitro* analysis

## Abstract

The emergence of the SARS-CoV-2 virus caused the COVID-19 outbreak leading to a global pandemic. Natural substances started being screened for their antiviral activity by computational and *in-vitro* techniques. Here, we evaluated the anti-SARS-CoV-2 main protease (M^pro^) efficacy of ^©^Rutan, which contains five polyphenols (R5, R6, R7, R7’, and R8) extracted from sumac *Rhus coriaria* L. We obtained three fractions after large-scale purification: fraction 1 held R5, fraction 2 consisted of R6, R7 and R7’, and fraction 3 held R8. *In vitro* results showed their anti-M^pro^ potential: IC_50_ values of R5 and R8 made 42.52 µM and 5.48 µM, respectively. Further, we studied M^pro^-polyphenol interactions by *in silico* analysis to understand mechanistic extrapolation of Rutan binding nature with M^pro^. We extensively incorporated a series of *in silico* techniques. Initially, for the docking protocol validation, redocking of the co-crystal ligand GC-376* to the binding pocket of M^pro^ was carried out. The representative docked complexes were subjected to long-range 500 ns molecular dynamics simulations. The binding free energy (BFE in kcal/mol) of components were calculated as follows: R8 (−104.636) > R6 (−93.754) > R7’ (−92.113) > R5 (−81.115) > R7 (−67.243). *In silico* results of R5 and R8 correspond with their *in vitro* outcomes. Furthermore, the per-residue decomposition analysis showed C145, E166, and Q189 residues as the hotspot residues for components contributing to maximum BFE energies. All five components effectively interact with the catalytic pocket of M^pro^ and form stable complexes that allow the estimation of their inhibitory activity. Assay kit analyses revealed that Rutan and its components have effective anti-SARS-CoV-2 M^pro^ inhibitory activity.

## 1 Introduction

As coronavirus infection became a global threat, different sources were searched for their anti-inhibitory activity. Among the inhibitory compounds investigated, polyphenols stood out for their high antiviral activity, which was attributed to their ability to inhibit the activity of the M^pro^. Phenolic compounds are considered to be the most promising among lower molecular weight compounds for their antiviral activity. The inhibitory nature of phenolics over SARS-CoV-2 is often linked with their influence on M^pro^ as well as spike proteins resulting from hydrogen and non-hydrogen bond interactions (Arunkumar et al., 2022). Compared to other compounds, phenolics showed lower mean values for the IC_50_ value due to their potent anti-M^pro^ activity (Yang et al., 2022). M^pro^ is a 33.8 kD protein that cleaves polyproteins at minimum 11 conserved sites (Ren et al., 2013; Jin et al., 2020) The specificity of M^pro^ is linked with cleavage around glutamine residue which is not found among human proteases (Pang et al., 2023). Thus, low toxicity of the M^pro^ inhibitors to host cells can be claimed.

Among natural substances, compounds that have phenolic rings deserve a special attention that could be reveal anti-M^pro^ activity (Pang et al., 2023). Polyphenols are suggested as effective inhibitiors of M^pro^ (Ahmad et al., 2024). Epigallocatechin-3-gallate (EGCG) is a polyphenol that was one of the earliest established inhibitors of the SARS-CoV-2 resulting from M^pro^ inhibition (Park et al., 2021). This compound, found as the main polyphenol in green tea (Graham, 1992), has been shown to reduce the enzymatic activity of HCoV-OC43 and HCoV-229E and is therefore considered a potential inhibitor of coronavirus replication (Jang et al., 2021). Theaflavin, another active phenolic compound found in black tea, has also been reported to have anti-M^pro^ activity (Jang et al., 2020). *In vitro* testing showed no significant differences in their inhibitory activity, with IC_50_ values of 16.53 µM for EGCG and 14.95 µM for theaflavin. In another study, tannic acid and 3-isotheaflavin-3-gallate were found to have IC_50_ values of 3 µM and 7 μM, respectively, indicating their potential as antiviral compounds. The authors report the higher antiviral potential of Puer and black teas compared to green ones (Chen et al., 2005). Processing conditions of black tea were investigated to enhance the content of theaflavin-3-3′-di-O-gallate in order to develop tea-based antiviral properties (Paiva et al., 2022). A more recent paper reported the inhibitory activity of several phenolics based on molecular docking and molecular dynamics (MD) simulations (Bahun et al., 2022). Among the twenty compounds tested, including flavonoids, curcuminoids, phenolic acids, and other polyphenols, quercetin, ellagic acid, curcumin, EGCG, and resveratrol showed the highest activity. Their IC_50_ values ranged from 11.8 to 23.4 µM dose. Additionally, by using computational biology approaches, geraniin isolated from *Geranium thunbergii* was reported as another potential inhibitor of this enzyme (Yu et al., 2022). Computational analyses of substances on viral proteins were effectively used to suggest their possible mechanisms of action (Singh and Purohit, 2024). *In silico* analyses on barrigenol, kaempferol, and myricetin suggested their potential as nonstructural Nsp15 protein of SARS-CoV-2 (Rashid et al., 2022; Otter et al., 2023; Sharma et al., 2021). Sings et al. screened the inhibitory properties of several curcumin derivatives on Nsp15 SARS-CoV-2 and reported potential ones (Singh et al., 2022). In another work, two of them were found to be effective inhibitors of RNA-dependent RNA polymerase (RdRP)-RNA complex (Singh et al., 2021). Inhibitory activity of tea-derived phenolics over M^pro^ was reported as another possible target that contributes to their anti-SARS-CoV-2 property (Bhardwaj et al., 2021).

M^pro^ was earlier reported as potential target to control coronavirus. The authors demonstrated the highest 3.7 µM IC_50_ value of broussoflavan, a prenylated quercetin derivative, among ten phenolics isolated from *Broussonetia papyrifera* (Park et al., 2017). Thus, prenylation was determined to double the inhibitory activity of quercetin (IC_50_ 8.6 µM). The IC_50_ values of broussochalcone A and B, differing by OH group presence, made 11.6 and 9.2 µM doses, respectively. Derivatives of darunavir were determined as SARS-CoV-2 M^pro^ inhibitors based on fluorescence resonance energy transfer (FRET) that were supported by molecular docking (Ma et al., 2022). Two of the studied compounds were further selected after *in vitro* analysis for designing inhibitors possessing higher inhibitory activity. Phenolic compounds isolated from brown marine algae *Ishige Okamurae* demonstrated high antiviral activity due to their ability to inhibit M^pro^ (Nagahawatta et al., 2022). Ishophloroglucin A was defined as the most potential one that inhibited the enzymic activity of M^pro^ and papain-like protease.

Further analysis in this discipline reported phenolics as the inhibitors of M^pro^ dimers. Acacetin 7-O-neohesperidoside, termed fortunellin, was found to be a potent inhibitor of M^pro^ dimer (Panagiotopoulos et al., 2021). *In silico* results were also confirmed by *in vitro* tests for a few phenolics: fortunellin, apiin, and rhoifolin. Three flavonoids such as baicalin, rutin, and glycyrrhizic acid were determined as potential antiviral agents against COVID-19 due to their inhibitory activity over M^pro^ after docking and molecular dynamics analysis (Patil et al., 2021). Pomegranate peel extract was reported as another source of potential antiviral agents against COVID-19. The alcohol extract containing polyphenols such as punicalagin, gallic acid, and ellagic acid was concluded to be an efficient means due to their involvement in many processes, including the inhibition of M^pro^ activity (Tito et al., 2021). Phenolics are indeed potential agents against SARS-CoV-2. Quercetin, for instance, was concluded as a possible therapeutic at the early stages of COVID-19 infection in a randomized clinical trial (Di Pierro et al., 2022).

**FIGURE 1:**
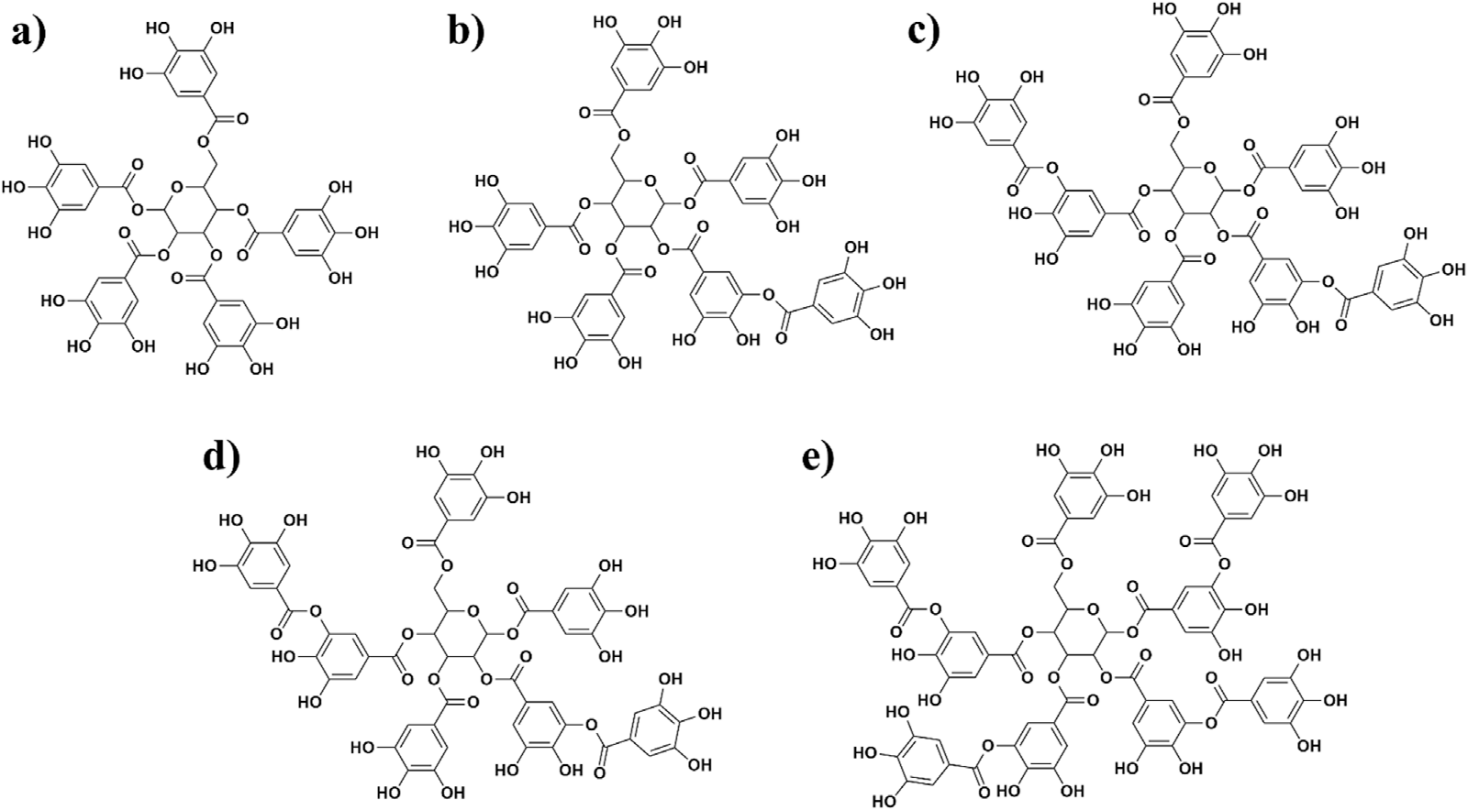
Structures of five polyphenols extracted from sumac *Rhus coriaria* L. that were investigated in this study [**(A)** = R5; **(B)** = R6; **(C)** = R7; **(D)** = R7’, and **(E)** = R8].

Sumac *Rhus coriaria* L plant polyphenols are mainly composed of gallic acid derivatives (Figure 1), and there are limited studies on their antiviral activity. Our group has previously examined the mixture of polyphenols, mainly tannin-like compounds, extracted from sumac *R. coriaria* L. for anti-influenza activity (Zhamollitdin Fazlitdinovich et al., 2020). The results showed remarkable potency for anti-influenza activity and leading to the commercialization under the name ^©^Rutan as an antiviral drug in the local market. Subsequently, during the pandemic, researchers studied anti SARS-CoV-2 activity of Rutan and observed high inhibitory efficacy against virus in infected cells during clinical trials (Salikhov et al., 2023). Toxicological analysis showed no drug accumulation in mice organs. Besides, no acute or chronic drug toxicity was observed; LD_50_ in rats and mice made >5,000 mg/kg when administered intragastrically. Pharmacological studies revealed no death when the drug was given at 2,000 mg/kg in 40 mice that aligned with no effects on orienting response and spontaneous motor activity. Moreover, no allergic reactions were observed when given at 25 mg/kg dose to guinea pigs (Salikhov et al., 2023). Five major compounds of polyphenolic nature in the Rutan composition–R5, R6, R7, R7’, and R8 are shown below in Figure 1. While various natural products, such as flavonoids and alkaloids, have been investigated for their potential inhibitory effects against SARS-CoV-2, many exhibit limitations such as poor bioavailability or cytotoxicity at effective concentrations (Xu et al., 2023). Sumac-derived polyphenolic compounds, however, represent a relatively unexplored class of natural inhibitors. These compounds are not only rich in antioxidant properties but also demonstrate favorable pharmacokinetics, making them promising candidates for therapeutic application. This study focuses on these compounds to explore their potential in inhibiting the SARS-CoV-2 main protease (M^pro^) through a combination of computational and experimental approaches.

Early analysis of Rutan on M^pro^ activity revealed concentration-dependent inhibition of the enzyme. A 17.7% inhibition rate of the enzyme by 0.5 µM dose of the mixture reached 64.4% rate by 5 µM dose (Salikhov et al., 2023). In this work, we aimed to explore possible mechanistic behavior of M^pro^-ligand interactions of Rutan by computational methods accompanied with *in-vitro* analysis of each fraction of Rutan components, namely, R5, R8 individually, and R6 + R7 + R7’ mixture (Figure 1), whether the compounds exhibit higher anti-M^pro^ activity alone or in complex.

## 2 Materials and methods

### 2.1 Preparation of M^pro^ and Rutan compounds for docking analysis

**FIGURE 2:**
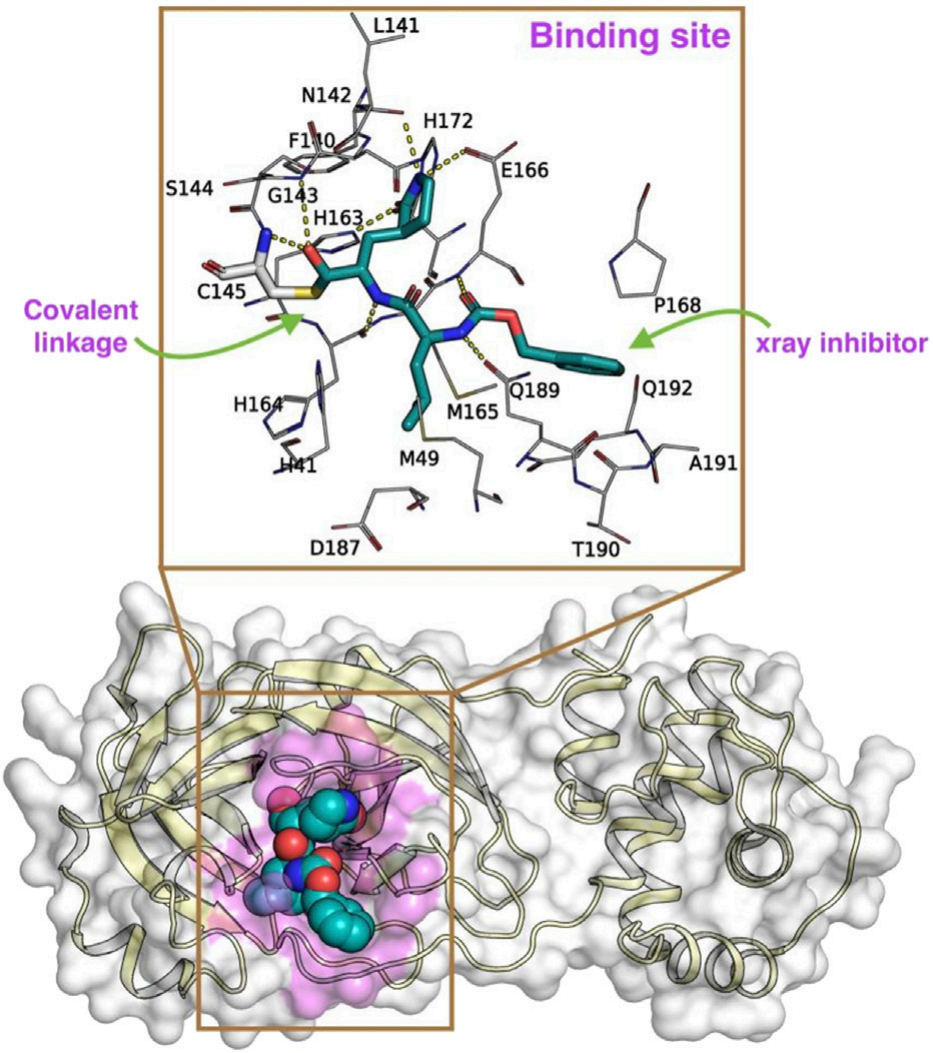
The x-ray crystal structure of SARS-CoV-2 M^pro^ [6WTT (Ma et al., 2020), represented in a cartoon format with the molecular surface highlighted in pale yellow and while, respectively] covalently bound to an inhibitor GC-376 (represented in spheres; carbon in marine blue; nitrogen in blue; and oxygen in red) used in this study. Here, the binding site of the M^pro^ is highlighted in pink color over the molecular surface. The magnified image highlights the (i) covalent linkage (green arrow) of the GC-376 inhibitor (represented in sticks in cyan color) to the C145 residue and (ii) other surrounding residues involved in M^pro^-Rutans complex formation. The yellow dotted lines represent the polar interactions between the GC-376 inhibitor and the surrounding residues of M^pro^.

The crystallographic structure of the SARS-CoV-2 M^pro^ complexed with inhibitor GC-376 (PDB ID: 6WTT, resolution of 2.15 Å; chain A) was retrieved from the Protein Data Bank (PDB at https://www.rcsb.org/structure/6WTT; Figure 2) (Ma et al., 2020). In this present investigation, we extensively utilized the Maestro suite 2020-4 (Schrödinger Inc. NY, United States) and the protocol used in our previous study (Marimuthu et al., 2021). To ensure the effective utilization of this suite, it is imperative to fix any pre-existing crystallographic artifacts that the M^pro^–GC-376 complex may contain. Moreover, this preparation step was crucial to enable subsequent investigations.

Consequently, the M^pro^ complexed with GC-376 underwent immediate preprocessing using default parameters, specifically employing the *Protein Preparation Wizard* (Sastry et al., 2013). This involved assigning bond orders, adding polar hydrogens, eliminating crystal waters, addressing missing side-chain residues, and establishing protonation states for charged residues (including Asp, Glu, Arg, Lys, and His) using PROPKA. Subsequently, the complex was subjected to minimization using the OPLS3 forcefield (Harder et al., 2016), maintaining a physiological pH of 7.2 with the assistance of Epik (Shelley et al., 2007; Greenwood et al., 2010). As a result of this thorough minimization process, the M^pro^–GC-376 complex was predicted to retain the charged residues in their native state, and H163 in ε, H164 in δ, H172 in a positive state, while H41 and C145 residues are in a neutral state.

**FIGURE 3:**
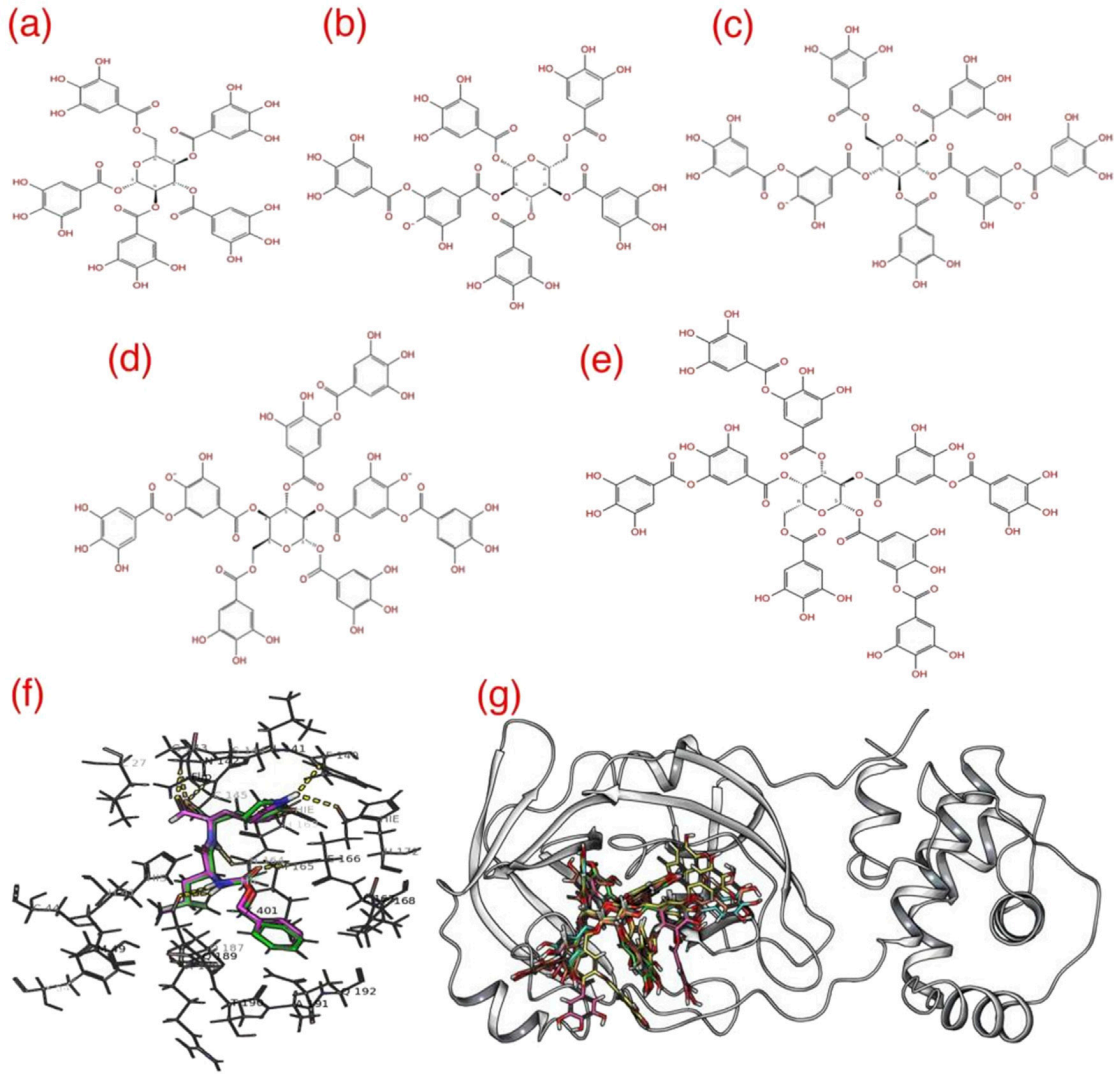
The 2D representation of five different Rutan **(A–E)** compounds used in this study. **(F)** The GC-376* redocked (pink) over the native GC-376 (green) showing reproducibility and **(G)** superposition of docked conformations of 5 Rutan compounds (R5 = yellow; R6 = green; R7 = pink; R7’ = cyan, and R8 = blue) inside the M^pro^ binding pocket. The yellow dotted lines highlight the polar interactions between GC-376* and the surrounding residues.

Subsequently, to create a molecular 2D representation of the five Rutan compounds – 1,2,3,4,6-penta-O-galloyl-β-D-glucose (R5), Hexa-O-galloyl-β-D-glucose (R6), Hepta-O-galloyl-β-D-glucose (R7), Octa-O-galloyl-β-D-glucose (R7’), Nona-O-galloyl-β-D-glucose (R8) (Salikhov et al., 2023) (Figures 3A–E), the “2D Sketcher” panel within Maestro was employed. These compounds underwent further processing, including the addition of any missing hydrogen atoms, correct assignment of formal charges, generation of plausible molecular configurations based on default ionization and tautomeric states, and ultimately convertion into their 3D representations using the OPLS3e (Roos et al., 2019), force field using the *LigPrep* module (Schrödinger Release 2020-4: LigPrep, Schrödinger, LLC, New York, NY, 2020). The resulting compounds, featuring low-energy conformational poses, were suitable and used for further investigation. The binding pocket analysis reveals that the co-crystallized ligand forms a covalent bond with the thiol group of the C145 residue that is well buried within the binding pocket of the M^pro^.

In order to carry out effective docking calculations, it is highly required to execute a redocking protocol, i.e., performing a docking run with the co-crystallized ligand on itself, which eventually exhibits the reliability and reproducibility of the crystal ligand conformation of the docking protocol planned on execution. Towards that, as the *Glide* program does not allow a covalently linked ligand to perform docking runs, a modified version of GC-376, referred to as “GC-376*” was created by manually breaking the native covalent linkage (Halgren et al., 2004; Friesner et al., 2004). Given the well-defined nature of the enzyme’s binding pocket, we opted to utilize monomeric conformation for our investigation, considering it to be a reliable approach, which has also been reported in previous studies (Choudhary et al., 2020; Wang, 2020; Mengist et al., 2021; Weng et al., 2021). As such, we believe that the methodology employed in our study offers a reasonable estimation of binding events and the relevant surrounding residues. The resulting M^pro^–GC-376* complex underwent a secondary round of minimization, following the previously outlined procedures. Subsequently, GC-376*, devoid of any local structural clashes or improper bond orders, was employed to establish a grid using the *Grid generation panel* available in Maestro, a prerequisite to conducting the Glide redocking process. Initially, the grid was generated by centering it on GC-376*, effectively encompassing the binding pocket enveloped by amino acid residues H41, C44, M49, Y54, F140, L141, N142, G143, S144, C145, H163, H164, M165, E166, L167, P168, H172, D187, R588, Q189, T190, A191 and Q192 of M^pro^. During the grid generation, process the additional parameters such as (i) assessing input ring conformations, (ii) favoring intramolecular hydrogen bonds, and (iii) improving the alignment of conjugated–π groups, while the remaining parameters were maintained at their default settings.

#### 2.1.1 Molecular docking

Next, to ascertain the stability of the crystal ligand’s with M^pro^, we conducted a redocking process centering GC-376* and the grid that was built previously. Throughout this procedure, default parameters were strictly followed, which involved assigning the van der Waals radii of nonpolar ligand atoms to 0.8, setting a partial charge cutoff of 0.15, and employing full-flexible docking. The docking operation utilized the Glide program accessible in Maestro (Friesner et al., 2004; Halgren et al., 2004), by specifying the *extra-precision (XP)* mode to obtain higher accuracy (Friesner et al., 2006). Additionally, the post-docking minimization was carried out on all generated poses obtained from the previous docking calculation. Subsequently, the top ten conformations for each ligand were selected for further analysis.

Following the insights gained from the redocking procedure, we proceeded to conduct the actual docking calculations for the five different Rutan compounds. For this purpose, all the preprocessed 3D conformations of the Rutan compounds, which were generated using the *LigPrep* calculations (740), were chosen. These selected conformations were then subjected to the docking procedure, utilizing the previously established grid and the predefined Glide parameters. The identification of the best docking hits was based on a comprehensive evaluation, considering the following criteria: (i) graphical investigation on the binding site of M^pro^, (ii) binding mode comparison with crystal ligand; i.e., comparison of the binding modes of the docked ligands with that of the crystal ligand to gauge their similarity and accuracy. (iii) glide scores: the glide scores obtained from the docking output are an integral part of the docking calculations and were utilized as a quantitative measure to rank and evaluate the binding affinities of the inhibitors. (iv) bonded and non-bonded interactions: detailed scrutiny of bonded (covalent) and non-bonded (non-covalent) interactions between the ligands and the receptor was performed to gain insights into the stability and specificity of the binding. These multi-faceted assessments allowed us to identify and select the most promising docking outputs for further analysis.

### 2.2 System setup for molecular dynamics simulations

To substantiate the binding interactions of the docked complexes, we employed Molecular Dynamics (MD) simulation techniques, following a protocol consistent with our prior research studies (Marimuthu et al., 2021; Perumal et al., 2022). To execute this process, we applied five representative holo complexes, (Rutan R5 to R8) that are bound within the binding pocket of M^pro^. These complexes were then subjected to the *system builder* panel in Maestro, where we constructed five independent simulation systems using the simulation parameters as detailed in Table 1.

**TABLE 1:** The simulation protocol involved in conducting long-range simulations for all five different M^pro^-Rutan complexes using Desmond, available in Maestro (Bowers et al., 2006).

Simulation protocol	
MD program	Desmond
Box type	Orthorhombic
Distance from solute surface	10 Å
Force Field	OPLS3e
Timestep integration	2 fs
Temperature	300K
Borostat	Martyna-Tobias-Klein (Lippert et al., 2013)
Thermostat	Nose-Hoover (Evans and Holian, 1985)
PME cutoff	0.9 nm
Long range electrostatics	K-space Gaussian split Ewald (Shan et al., 2005)
Short-range non-bonded interaction	r-RESPA integrator (Masella, 2006)
vdW interaction monitoring	Uniform density approximation
H bond constraint	M-SHAKE integrator (Lambrakos et al., 1989)
Neutralizer	NaCl
Salt concentration	0.15 nM
Solvent type	TIP3P
Multi-stage equilibration	
Brownian Dynamics NVT	T = 10 K, small timesteps were utilized during 100 ps simulations along with restraints on solute heavy atoms
NVT	T = 10 K, small timesteps were again employed during 12 ps simulation by applying restraints on solute heavy atoms
NPT	T = 10 K, and positions restraints were applied on solute heavy atoms, during 12 ps simulations
	further, restraints were again applied on solute heavy atoms during 12 ps simulations
	finally, position restraints were removed and performed 24 ps simulations
Production runs	500 ns
Replicates	5

#### 2.2.1 Trajectory analysis

The analysis of all trajectories obtained from the molecular MD simulations was conducted using the built-in modules, such as *Simulation Quality Analysis* and *Simulation Interaction Diagram*, available in Maestro. Furthermore, we calculated the overall count of intermolecular hydrogen bonds formed between the ligands, and the binding site residues of the M^pro^ was computed using the *Interaction Count* module.

#### 2.2.2 Binding free energy estimation using MM-GBSA approach

For the estimation of Binding Free Energy (BFE) values pertaining to the M^pro^–Rutan compounds, we utilized the *Prime thermal_mmgbsa.py* (Jacobson et al., 2004) script, an integral component of the Maestro suite. The script employs the Molecular Mechanics-Generalized Born Surface Area (MM-GBSA) method to compute the BFE values for the given M^pro^–Rutan complexes using the OPLS3e force field and the Variable Solvent Generalized Born (VSGB) solvation model, using default parameters. The OPLS3e force field integrates the CM1A-BCC charge model, which combines Cramer-Truhlar CM1A charges with extensive Bond Charge Correction (BCC) parameters. Additionally, default dielectric constant cutoff values (1 for the solute and 80 for the solvent) were used. Throughout the computations, the program provided estimations for various energy components, encompassing H-bond interactions, van der Waals forces, Generalized Born (GB) solvation energies, Coulombic interactions, π-π stacking interactions, lipophilic interactions, and self-contact interaction terms. These calculations were conducted separately for the M^pro^, Rutans, and the M^pro^–Rutan complexes.

$$∆G_{\text{bind}}=E_{\text{Complex}}\;–\;E_{\text{Ligand}}\;–\;E_{\text{Receptor}}$$

Furthermore, the ∆G values can be subdivided into E_lipophilic_, E_electrostatics_, and E_vdW_ interaction components,

Where E_electrostatics_ = E_H bond_ + E_coloumb_ + E_GB_solvation_ and E_vdW_ = E_vdW_ + E_π-π_ + E_self-contact_ and E_lipophilic_.

The MM-GBSA-based energy estimation method in Maestro does not include the calculation of conformational entropy, thus, the entropic values were not determined.

### 2.3 *In vitro* method: M^pro^ bioassay and mechanism

The assay kit was purchased from BPS Bioscience. In short, the mechanism of the coronavirus M^pro^ test is based on the inhibition of the protease activity of the M^pro^ that cleaves a specific peptide substrate, in which fluorescence is produced. In the presence of an inhibitor, the M^pro^ is blocked, and non-cleaved peptide substrates do not produce or produce low fluorescence. The fluorescence intensity is correlated, i.e., directly proportional to the percentage inhibition.

The experiment was carried out according to the manufacturer’s protocol. For this assay, M^pro^ (4 ng/μL, i.e., 120 ng) was first treated with 10 μL of the test sample and mixed with M^pro^ substrate (diluted in 10 μL assay buffer). The microplate was left overnight at room temperature, after which the fluorescence was measured by exciting at 360 nm and detecting at 460 nm.

The inhibition effect on M^pro^ was calculated using the formula:

$$Inhibition\;\left(\%\right)=\left[1-\frac{{FL}_{with\;inhibitor}-{FL}_{blank}\;}{{FL}_{without\;inhibitor}-{FL}_{blank}}\right]\times 100$$

### 2.4 Stability test of Rutan and components

The stability of the components was checked in various pH media ranging from pH 5 to 9: 0.02 M sodium acetate buffer was used for pH 5.0; 0.02 M sodium phosphate buffer was used for solutions with pH 6.0 and 7.0; 0.02 M ammonium bicarbonate buffer was used for preparing solutions with pH 8.0-9.0. For stability analysis, 2.0 mg of Rutan substance was dissolved in 2 mL of appropriate buffer. The prepared solutions were incubated at 37°C for 1 h and 24 h. Then, their stabilities were checked using HPLC, Agilent Technologies series 1,200 s equipped with DAD detector, and column 4.6 × 250 mm Zorbach Eclipse C18, 5 µm. A: 0.1% trifluoroacetic acid (pH 2.5), B: CH_3_CN. We used the following gradients: 15%–3 min; 25%–17 min; 60%–8 min; 15%–2 min.

## 3 Results and discussion

### 3.1 Molecular docking

Evaluation of GC-376* redocking and Rutan compounds to the binding pocket of M^pro^: To validate our docking protocol, we conducted a redocking process by subjecting the modified GC-376* to its native state GC-376, and subsequently compared the outcomes by superimposing the docking results (Figure 3F). The analysis reveals a remarkable similarity between the native and redocked conformations. Furthermore, our results underscore that the redocked conformation adeptly preserves all crucial interactions, including vital polar contacts.

**FIGURE 4:**
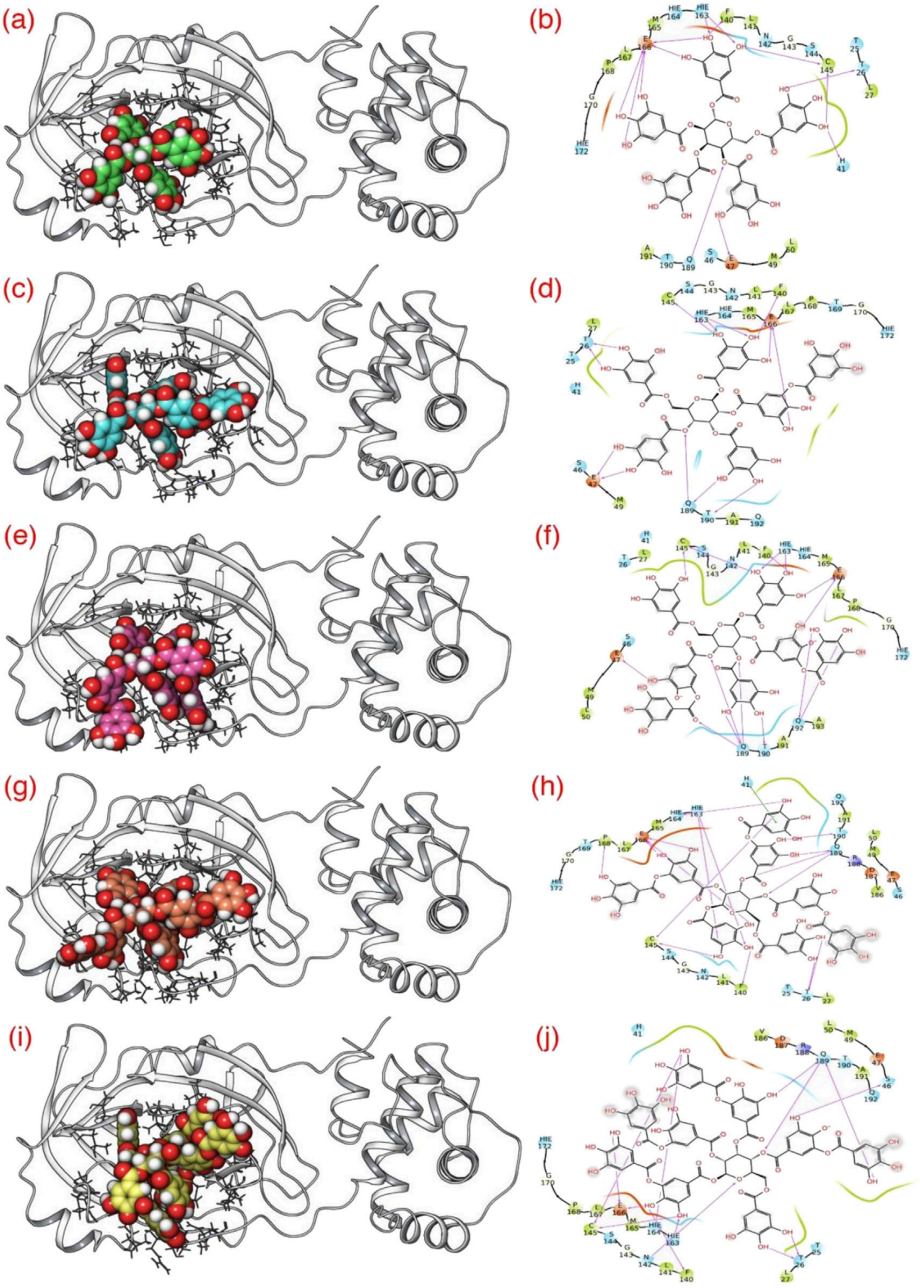
Docking of five different Rutan compounds (spheres) to the binding pocket of M^pro^: **(A, C, E, G, I)**. The 3D and **(B, D, F, H, J)** 2D representation of Rutan compounds interacting to M^pro^ binding pocket. The pink arrows represent the H-bond interactions with specific residues inside the binding pocket of M^pro^, while the direction of the arrows represents the donor and acceptor atoms involved.

Consequently, for the current study, we adhered to the same docking parameters, employing the Glide program for all five Rutan compounds. Additionally, our comprehensive examination of the binding modes of all Rutan compounds reveals that they occupy the same binding pocket as GC-376*, engaging in analogous interactions (Figures 3G, 4; Table 2). Overall, these findings affirm that all five Rutan inhibitors effectively interact to the same binding pocket of M^pro^, collectively covering a significant interaction surface. While molecular docking provides valuable insights into potential binding affinities, it is important to note its semi-quantitative nature. The scoring algorithms influence docking scores and may not fully account for dynamic protein-ligand interactions, particularly in flexible systems such as SARS-CoV-2 M^pro^. To mitigate these limitations, we conducted molecular dynamics simulations, which allowed us to study the stability of the binding interactions over time. However, force field limitations in MD simulations, such as approximations in protein flexibility and solvation effects, can also influence the accuracy of the results. Experimental validation, including enzymatic assays or co-crystallization studies, is essential to confirm the inhibitory effects observed *in silico*.

**TABLE 2:** List of residues of M^pro^ interacting with five different Rutan compounds.

Ligands	Residues–4Å distance	Polar	Electrostatic	Hydrophobic
R5	T25, T26, L27, H41, S46, E47, M49, L50, F140, L141, N142, G143, S144, C145, H163, H164, M165, E166, L167, P168, G170, H172	T26, H41, C145, H163	E47, E166, Q189	L27, M49, L50, F140, L141, G143, C145, M165, L167, P168, A191
R6	T25, T26, L27, H41, S46, E47, M49, F140, L141, N142, G143, S144, C145, H163, H164, M165, E166, L167, P168, T169, G170, H172, Q189, T190, A191, Q192	T26, F140, C145, H163, T190	E47, E166, Q189	L27, M49, F140, L141, G143, M165, L167, P168, G170, A191
R7	T26, L27, H41, S46, E47, M49, L50, F140, L141, N142, G143, S144, C145, H163, H164, M165, E166, L167, P168, G170, H172, Q189, T190, A191, Q192, A193	F140, C145, H163, T190	E47, E166, Q189, Q192	L27, M49, L50, F140, L141, G143, C145, M165, L167, P168, G170, A191, A193
R7’	T25, T26, L27, H41*, S46, E47, M49, L50, F140, L141, N142, G143, S144, C145, H163, H164, M165, E166, L167, P168, G170, H172, V186, D187, R588, Q189, T190, A191, Q192	T26, F140, C145, H163, H164, P168, T190	E166, Q189	L27, M49, L50, F140, L141, G143, C145, H163, H164, M165, L167 G170, V186, A191
R8	T25, T26, L27, H41, S46, E47, M49, L50, F140, L141, N142, G143, S144, C145, H163, H164, M165, E166, L167, P168, G170, H172, V186, D187, R588, Q189, T190, A191, Q192	T26, S46, F140, C145, H163, H164	N142, E166, Q189	L27, M49, L50, F140, L141, G143, C145, H163, H164, M165, L167 G170, V186, A191

### 3.2 Molecular dynamics simulations

To assess the stability of the five docked complexes and gain an in-depth understanding of the impact of different inhibitors with M^pro^ over time, we have built a series of five individual MD simulation systems. Subsequently, each system was subjected to 500 ns independent production runs. Later, the simulation outputs were rigorously examined for their structural stability using the fully automated panels such as *Simulation Quality Analysis*, *Simulation Interaction Diagram*, and *Interaction count* in Maestro-GUI, and the results were plotted.

#### 3.2.1 Structural stability analysis for the M^pro^-Rutan compounds

**FIGURE 5:**
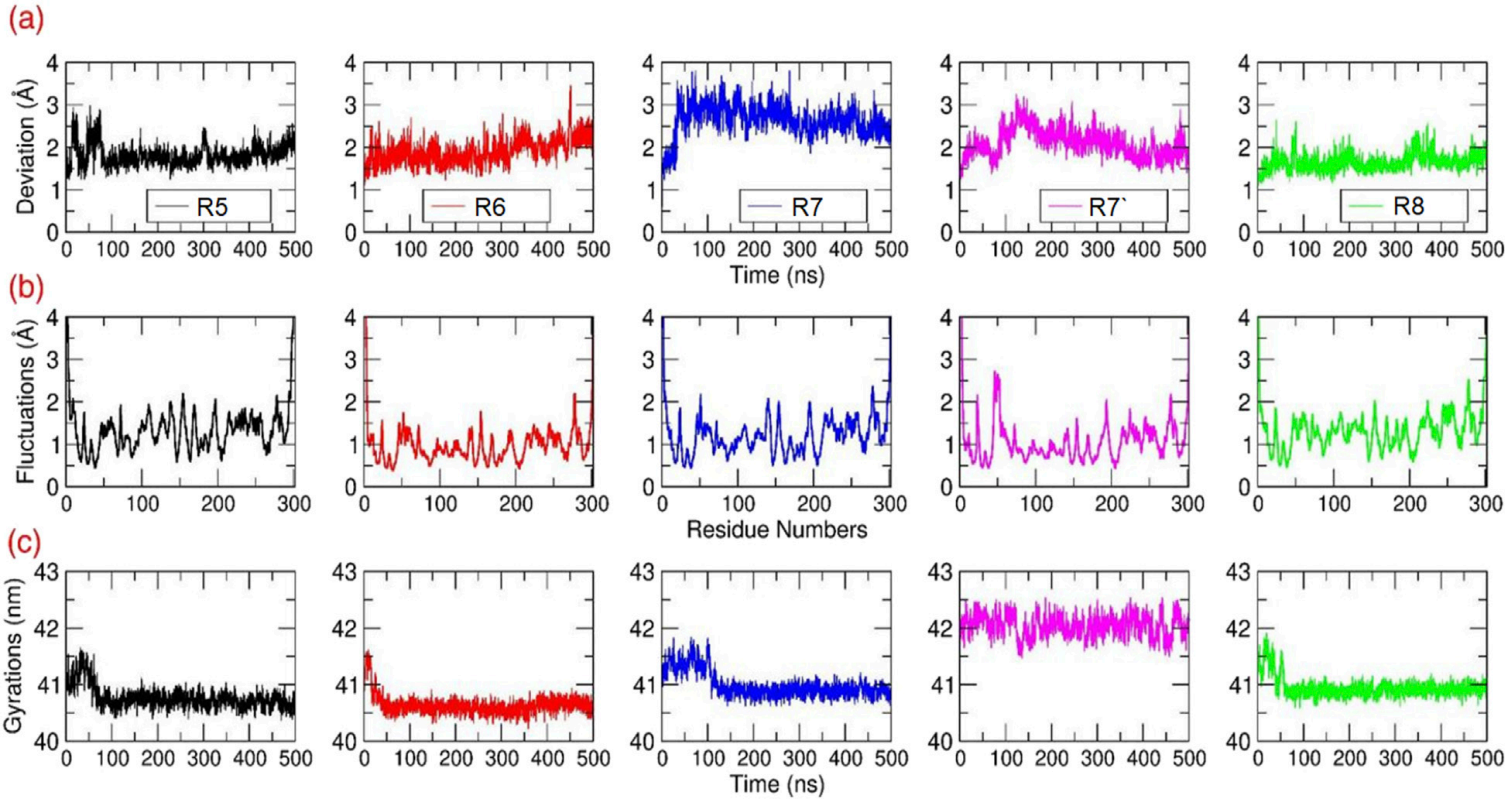
Structural Analysis: The **(A)** RMSD **(B)** RMSF and **(C)** Rg values in Å were calculated for all the Cα atoms of M^pro^ bound to different Rutans based on the 500 ns MD simulations.

Root Mean Squared Deviation (RMSD in Å) estimation for the M^pro^–Rutan compounds: The structural deviations were computed for all the Cα atoms of each M^pro^-Rutan complex throughout the MD simulation (Figure 5A). The RMSD graphs illustrate that all M^pro^-Rutan complexes swiftly reached an equilibrium phase following an initial relaxation period, showing deviations ranging from approximately 1.75–2.65 Å from their starting structures. Specifically, the RMSD graphs for M^pro^ complexed with R5, R6, and R8 compounds exhibit a consistent equilibrium phase, demonstrating a stable equilibration phase throughout the simulation, maintaining the RMSD value of ∼1.75 Å, each. This indicates that when R5, R6, and R8 compounds bind to M^pro^, they do not induce substantial conformational changes compared to their initial states. Similarly, the rmsd values for the M^pro^-R7 complex show a stabilized state only after experiencing an initial fluctuation phase for ∼60 ns, later maintaining an overall RMSD value of ∼2.5 Å. However, the RMSD plot for the M^pro^-R7’ complex indicates a conformational deviation occurring during earlier in the simulation period, from 0 to 170 ns, followed by reaching an equilibrium phase, sustaining rmsd values between ∼1.8 and 2.3 Å. Overall, the RMSD analysis across the five distinct simulation outputs involving M^pro^-Rutan complexes suggests that the binding of Rutans to M^pro^ does not significantly alter the overall protein conformation. Hence, the frames obtained from the final 200 ns of the MD simulations are considered reliable and suitable for further analysis.

Root Mean Square Fluctuations (RMSF in Å) estimation for the M^pro^–Rutan complexes: The structural flexibility of all the Cα atoms present in each M^pro^-Rutan complexes were computed during the MD simulation (Figure 5B). Overall, the RMSF graphs displayed multiple segments of structural stability and flexibility in Cα atoms of the M^pro^ structure in the same trend. i.e., the M^pro^ exhibited (i) elevated fluctuations were observed at the loop and termini regions, as anticipated, and (ii) distinct differences in backbone fluctuations were evident among the inhibitors, especially at the binding site region. Additionally, the residues involved in the binding site region exhibited only a lower level of fluctuations for the compounds R5 to R8. This indicated that Rutans exhibiting weak binding affinity displayed moderate or weak interaction with the binding site residues, leasing to higher fluctuation compared to other complexes. Conversely, inhibitors with strong binding affinity demonstrated tight and stable interactions with the binding site residues, resulting in reduced or less fluctuation of M^pro^.

Radius of Gyrations (Rg in Å) estimation for the M^pro^–Rutan complexes: The Rg analysis from the MD simulation will provide comprehensive information on the change in the overall internal structure or the compactness of the macromolecule due to ligand binding. During the simulation, the overall compactness is measured based on rms-distance of all Cα atoms of M^pro^ from its center-of-gravity. In this study, the Rg values were extracted for all M^pro^-Rutan complexes from all the trajectories and plotted (Figure 5C). The Rg graph shows that from the starting initial time period until ∼100 ns, the M^pro^ experienced a minor fluctuation in all the systems. Subsequently, the M^pro^ maintained stability throughout the simulation until the end, with the stable Rg values between ∼40.5 and 41 nm for the systems R5-R7 and R8, respectively, whereas the M^pro^-R7’ maintains its Rg values at ∼42 nm. Overall, the Rg plot displays only a relatively minor change in the structural compactness during the simulation, which signifies that the binding of Rutans did not majorly alter the internal compactness of M^pro^.

#### 3.2.2 Polar contacts estimation between M^pro^ and Rutan compounds

One work has reported that the GC-376 inhibitor effectively occupied the binding pocket of M^pro^ (Ma et al., 2020). This binding pocket is encompassed by a network primarily composed of hydrophobic residues, prominently featuring L27, M49, L50, F140, L141, G143, C145, H163, H164, M165, L167 G170, V186, and A191. Moreover, their study revealed that inhibitor GC-376 engaged in interactions with C145 through bisulfite adducts, leading to the formation of covalent complexes characterized by a tetrahedral arrangement. Additionally, the GC-376 inhibitor was found to establish various other interactions, notably hydrogen bond interactions, with essential residues, including T26, S46, F140, C145, H163, H164, and electrostatic interactions with N142, E166, and Q189.

**FIGURE 6:**
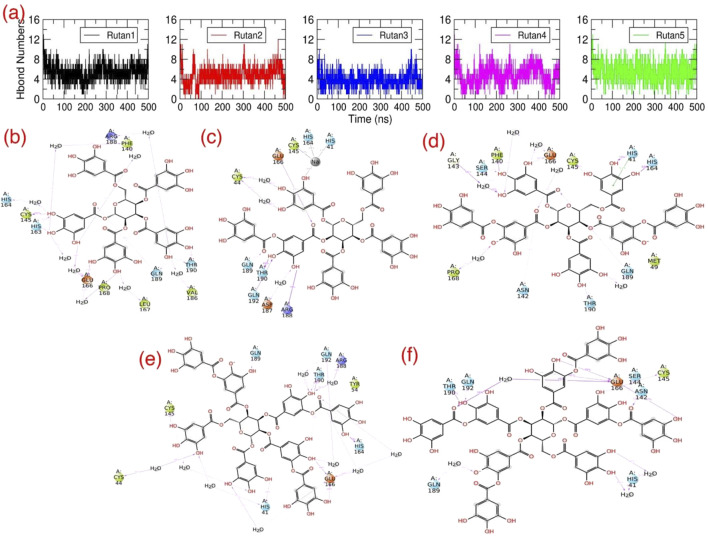
H bond interactions of Rutan compounds with M^pro^: **(A)** The total number of polar contacts and **(B–F)** 2D representations of polar interactions between M^pro^ and individual Rutan compounds over time. The occupancies of each polar interaction are presented in percentages.

Concurrently, in addition to the initial structural investigation, we also explored the H bond interaction pattern of the Rutan compounds with the binding pocket residues of M^pro^ towards the complex formation. The initial graphical assessment of MD trajectories focusing on the interaction between Rutans and M^pro^ underscores a robust interaction within the binding pocket. This interaction is characterized by a firm establishment of both hydrophobic and polar interactions, with the involvement of additional residues similar to the native crystal structure contributing to the complexity of the binding. Moreover, for a deeper insight into the stability of this complex, we systematically tracked the total number of polar interactions (Figure 6A) between M^pro^ and Rutans over the course of the simulations.

The graph shows that during the simulation, all five complexes have revealed a significant number of polar interactions with the surrounding residues to maintain the M^pro^-Rutan complexity (Figure 6A) that helped the compounds to stay intact within the binding pocket. In this aspect, the R5 and R8 compounds exhibited as high as (∼7–8) polar interactions, while the R6 and R7’ compounds exhibited relatively lesser polar contacts, i.e., ∼6 to 7, and the R7 compound exhibited much fewer (∼5–6) in comparison with other Rutans during the simulation. The 2D polar interaction graph (Figures 6B–F) reveals that E166 and Q189 residues of M^pro^ establish electrostatic interactions, while C145 and T190 contribute polar interactions with all Rutan compounds, whereas H41, H164, and Q192 establish polar interaction in most of the case, providing insights into the stability and frequency of these interactions.

In summary, these findings underscore the significant impact of polar interactions between the binding site residues of M^pro^ and the inhibitors on complex stability. This, in turn, results in elevated binding affinity values. Notably, complexes with higher binding affinity values exhibited a greater number of polar contacts, whereas those with lower binding affinity values displayed a reduction in such contacts. These insights are indispensable for the precise optimization and augmentation of the binding capabilities of a lead compound, particularly when targeting a receptor with significant activity initially detected during screening.

#### 3.2.3 Estimation of MMGBSA-based binding free energies for the M^pro^–Rutan complexes

The essential role played by M^pro^ in the SARS-CoV2 viral replication process underscores the urgent need to unravel its mechanistic intricacies and expedite the discovery of high-affinity inhibitors specifically tailored for M^pro^. To achieve this, we leverage advanced computational programs, both open-source and commercial, in association with the state-of-the-art high-performance computing systems. In our research, these computational tools facilitated rapid prediction of Binding Free Energies (BFEs) for a set of five distinct Rutan compounds, leveraging their three-dimensional molecular coordinates. This approach has the potential to streamline the design and evaluation of M^pro^ complexed with Rutan inhibitors, promising enhanced precision and efficacy in this process. Specifically, we extensively employed the *thermal_mmgbsa.py*—a built-in trajectory post-processing tool in Maestro—to predict BFE values for all five M^pro^–Rutan complexes, averaging results from five replicates (Table 3; Supplementary Table S1). Utilizing 100 snapshots from the last 200 ns of the MD trajectories, the predicted BFE values for these complexes are as follows: −81.11, −93.75, −67.24, −92.11, and −104.63 kcal/mol, respectively.

**TABLE 3:** Binding free-energy (kcal/mol) estimation obtained from the last 200 ns and an average of five replicates of MD simulations and its individual components using MMGBSA approach.

Interacting components	Rutan compounds				
	R5	R6	R7	R7’	R8
Coulomb	−46.805	−67.972	−17.540	−42.363	−41.817
Covalent	3.506	4.021	4.926	0.658	2.2482
H bond	−3.177	−4.584	−3.343	−4.571	−5.984
Lipo	−19.860	−18.332	−18.058	−18.461	−23.597
π-π Packing	−1.1002	−2.777	−1.880	−3.895	−4.091
Solv_GB	40.764	53.433	26.457	41.202	45.492
Solv_SA	−54.442	−57.541	−57.804	−64.684	−76.885
vdW	11.419	14.031	15.364	23.849	25.521
ΔG_MMGBSA_	**−81.115**	**−93.754**	**−67.243**	**−92.113**	**−104.636**

Average binding free energies show the mean values of five replicates for each compound-M^pro^ complex.

Apart from determining the total ΔG_bind_, the program also provides a detailed insights into various interaction components, encompassing Coulombic forces, covalent binding, hydrogen bonding, lipophilic interactions, π−π stacking, Solv_GB (Generalized Born solvation), solvation surface accessibility (Solv_SA), and van der Waals forces (Table 3). These individual interacting components hold immense potential for future applications, especially in the domain of novel drug discovery for M^pro^ inhibition. Moreover, these components offer an extensive understanding of the intricate binding process between Rutan and M^pro^. Consequently, the predicted ΔG^bind^ is explored for its constituent energy components, enriching our understanding of the underlying binding mechanisms.

Among these individual energy components, Coulombic, Lipophilic, and Solv_SA terms exclusively contribute the most favorable energies for complex formation, while the hydrogen bond component provides relatively less favorable energy across all Rutan compounds. Notably, these energy trends align with the total number of hydrogen bond analyses depicted in Figure 6. Conversely, the Solv_GB and vdW energy terms contribute higher unfavorable energies, while the Covalent energy terms contribute relatively less unfavorably to the R5, R6, R7, and R8 compounds, and negligibly unfavorably to the R7’ system. Overall, the analysis of individual energy components across all simulation systems involving M^pro^ complexed with multiple Rutan compounds highlights the substantial energies provided by the Coulombic, Lipophilic, and Solv_SA energy terms for complex formation.

#### 3.2.4 Per residue decomposition

In the current study, the per-residue decomposition (PRD) analysis was conducted using the Prime *thermal_mmgbsa.py* script, with the same number of frames and an average of five replicates used to compute the BFE values for five different Rutan compounds when bound to M^pro^ (Table 4 PRD). This decomposition analysis provides profound insights by showcasing the specific key residues at the interface that predominantly contributed to the BFE values due to Rutans binding. Further examination, particularly focused on the binding site residues of M^pro^, unraveled intricate variations in energy profiles within a diverse ensemble of hydrophobic and polar residues, shedding light on their distinctive roles in the binding process.

**TABLE 4:** The Per-Residue Decomposition (PRD) values extracted for the M^pro^ binding site residues obtained from the last 200 ns and an average of five replicates of MD simulations. The PRD values were extracted from total BFE values.

Residues	R5	R6	R7	R7’	R8
T25	−0.014	−0.021	0.065	−0.005	0.008
T26	−1.038	−1.065	−1.027	−1.010	−1.015
L27	−0.011	−0.018	0.120	−0.012	0.008
H41	−0.358	−0.659	−0.918	−1.113	−1.434
S46	−1.169	−1.018	−0.426	−0.940	−1.026
E47	−1.112	−1.177	−1.241	−1.869	−1.325
M49	−0.633	−0.198	−0.386	−0.387	−0.560
L50	0.299	0.143	0.718	0.094	0.335
F140	−0.164	−0.454	−0.069	−0.012	−0.292
L141	0.219	0.162	0.252	0.197	0.440
N142	−0.465	−0.927	−1.816	−0.673	−1.724
G143	−0.071	−0.194	−0.895	−0.200	0.137
S144	−0.064	−0.058	−0.235	−0.049	−0.194
C145	**−2.931**	**−2.950**	**−2.385**	**−2.011**	**−2.221**
H163	−1.052	−1.118	−1.502	−1.250	−1.334
H164	−0.399	−0.479	−0.068	−1.357	**−1.987**
M165	−0.423	−0.542	−0.106	−0.520	−0.031
E166	**−3.555**	**−3.131**	**−4.619**	**−3.099**	**−4.964**
L167	−0.305	−0.406	−0.708	−0.703	−0.920
P168	0.106	0.329	0.513	0.458	0.264
G170	−0.156	−0.135	0.376	0.026	−0.112
H172	−0.078	−0.231	−0.852	−0.062	−0.127
V186	0.096	0.192	−0.311	0.376	0.125
D187	0.408	0.605	−1.011	−1.195	−1.404
R588	−1.415	−1.098	−0.844	−1.168	−0.878
Q189	**−3.056**	**−2.901**	**−3.017**	**−3.928**	**−2.733**
T190	−1.002	−1.024	−1.028	−1.186	−1.552
A191	0.457	−0.012	0.915	0.942	0.431
Q192	−0.642	−0.627	−0.282	−0.87	−0.584

The key amino acid residues C145 (Cysteine 145), E166 (Glutamic acid 166), and Q189 (Glutamine 189) with the maximum contribution to the complex formation are given in bold.

While the strong binding affinities of Rutan compounds to the SARS-CoV-2 M^pro^ active site are encouraging, it is important to evaluate their potential off-target effects. Polyphenolic compounds are known to interact with various proteases and enzymes, which could lead to unintended biological consequences. For instance, flavonoids such as quercetin have been reported to inhibit proteases beyond their intended targets, potentially affecting normal cellular processes (Di Petrillo et al., 2022). To mitigate this concern, future studies should involve broader *in vitro* screenings against a panel of proteases to assess the specificity of Rutan compounds. Additionally, *in vivo* toxicity studies will be essential to evaluate their pharmacokinetic and pharmacodynamic profiles. By understanding the potential off-target interactions, we can design more selective derivatives to minimize adverse effects.

**FIGURE 7:**
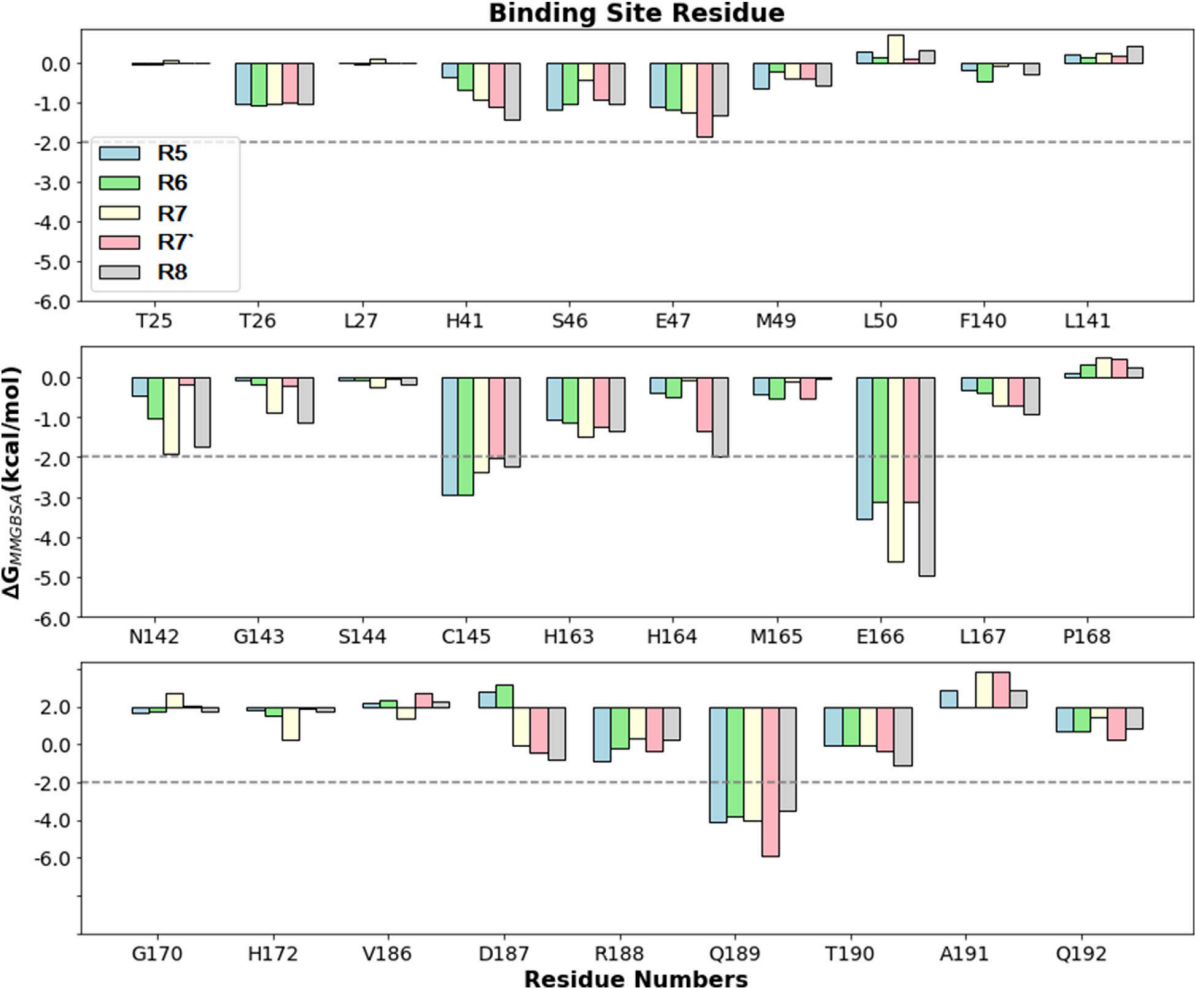
Comparison of PRD (kcal/mol) for different Rutan compounds bound to M^pro^ binding site residues. The −2 kcal/mol benchmark value is assigned to obtain the hotspot residues.

Here, the key residues with the maximum contribution to the complex formation (hot spot residues) are extracted from all binding site residues by assigning −2 kcal/mol (Table 4; Figure 7). Based on thiscriteria, the residues with maximum contributions for Rutan compounds are C145, E166, and Q189.

### 3.3 Stability of fractions

**FIGURE 8:**
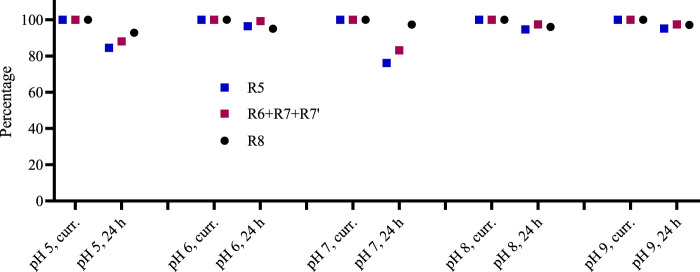
The stability of Rutan compounds was assessed at various pH conditions in freshly prepared solutions and after 24 h (n = 2, SD is less than 3% in all cases).

Polyphenols are low-acidic compounds, and acidic conditions do not affect their structures (Xiao, 2022). We applied pH conditions 5–9 and incubated for 24 h to investigate the stability of the Rutan and its components in various pH conditions (Figure 8). The obtained results indicated that all three fractions were stable at pH 8 and pH 9. We did not observe pH-dependent changes. Reversely, changes that were not observed in pH 6, pH 8, and pH 9 were found in pH 5 and pH 7. Noticeable degradation was observed for R5 in pH 5 and pH 7, around 15% and 25%, respectively. The mixture of R6 + R7 + R7’ showed 83% and 80% stability at pH 5 and pH 7 conditions for 24 h. Among the fractions, the R8 was found to be the most stable in all pH conditions. Overall, our study confirmed the stability of Rutan components in 5–9 pH conditions.

### 3.4 *In vitro* evaluation of M^pro^-Rutan complexity

**FIGURE 9:**
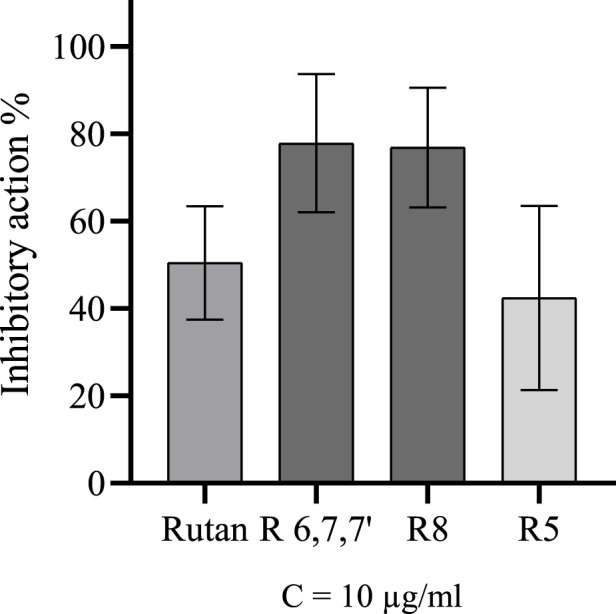
Inhibitory activity of Rutan and its components over M^pro^.

Rutan is a mixture of polyphenolic compounds, marked as R5, R6, R7, R7’, and R8. The mixture and its compounds separated individually R5 and R8, as well as the fraction containing R6, R7, and R7’, were tested for the M^pro^ inhibitory activity at 10 μg/mL concentration. The fraction consisting of the three components and compound R8 inhibited the enzyme 77.8% and 76.8% accordingly, while the Rutan substance and compound R5 resulted in 50.4% and 42.4% inhibition, respectively (Figure 9). Since the components R5 and R8 are pure compounds, their IC_50_ values were also calculated and equal to 42.52 µM and 5.48 µM accordingly. The difference between the IC_50_ values is positively correlated with the corresponding data in Figure 9.

The results of the *in vitro* M^pro^ inhibitory activity of Rutan and its components align with the findings of the *in silico* analysis conducted in this study. Consistent with the computational predictions the R8 component demonstrated the highest efficacy, while the fraction of R6, R7, and R7’ also exhibited significant inhibitory potential. The highest inhibition rate of M^pro^ by the fraction consisting of R6, R7, and R7’ can be linked with calculated ΔG_MMGBSA_ values of R6 and R7’, which were higher than the value of R5. The lowest *in vitro* inhibitory efficacy belonged to R5, which caused twice the lower inhibitory activity of the total phenolics in the composition of Rutan compared to R8 or the fraction.

According to Figure 9, compound R8 and the fraction holding R6, R7, and R7’ have almost 80% inhibitory activity, much higher than the inhibitory activity of R5. The computed binding free energies for R8, R6, R7, and R7’ were calculated to be −104.636, −93.754, −67.243, and −92.113 kcal/mol, respectively, which are not in complete correspondence with *in vitro* results. As the mixture of R6, R7, and R7’ exhibited 77.8% inhibition of M^pro^, their ΔG_MMGBSA_ values also should not be different from the ΔG_MMGBSA_ values of R8 theoretically. This discrepancy might be linked to the probable synergistic effect of R6, R7, and R7’ compounds (Salikhov et al., 2023). Their combined action could enhance their efficacy as potent M^pro^ inhibitors. However, further in-depth research is required to confirm and elucidate this synergistic mechanism.

Plant-based natural products, including flavonoids, alkaloids, and polyphenols, have been extensively studied for their antiviral potential against SARS-CoV-2. For example, quercetin, a well-known flavonoid, has shown moderate inhibitory effects on SARS-CoV-2 M^pro^, but its bioavailability and stability remain significant challenges (Xu et al., 2023). Similarly, berberine, an alkaloid, has demonstrated strong binding affinity in computational studies, yet its cytotoxicity limits its therapeutic application (Oner et al., 2023). In contrast, sumac-derived polyphenolic compounds, particularly Rutan, offer distinct advantages. These compounds exhibit strong binding affinities to SARS-CoV-2 M^pro^, as demonstrated in our molecular docking and dynamics studies, while maintaining low cytotoxicity, as supported by our *in vitro* assays. Additionally, the unique molecular structure of Rutan facilitates stable interactions with key residues such as C145 and E166, which are crucial for protease inhibition. This makes sumac polyphenols a promising class of natural inhibitors with potential for further development.

M^pro^ inhibitory activity of reference drug GC376 was equal to IC_50_ = 0.27 µM, which was several times more active than the Rutan or its components alone. It might be the suitable structure of GC376 for the protease pocket and covalent bond interactions with the Cys145 amino acid of the M^pro^ active pocket (PDB ID: 6WTT) (Ma et al., 2020). However, Rutan compounds deserve more attention because of their very low toxicity and side effects, even in high doses. Early clinical studies of Rutan tablets on randomly selected patients infected with SARS-CoV-2 demonstrated significantly lower post-COVID-19 manifestations after administration of 25 mg of Rutan to children and 100 mg drug to adults. Besides, significantly lower levels of C-reactive protein were attributed to Rutan administration in adults. The administered 100 mg dose to adults two times a day was found effective for patients with mild COVID-19 disease (Salikhov et al., 2023).

## 4 Conclusion

*The in vitro* results showed comparable M^pro^-inhibitory effects of the R8 and the mixture of R6, R7 and R7’ compared to R5. We established IC_50_ values of two polyphenolic compounds R5 and R8 towards M^pro^. The mechanism of action of M^pro^-Rutan components determined by computational methods affirmed that all five Rutan components formed a stable complex with M^pro^, effectively interacting with its binding pocket and covering a significant interaction surface. Notably, the binding of Rutans did not significantly alter the overall conformation or structural compactness of M^pro^ during the simulation. BFE calculations of compounds revealed that R8, R6, and R7’ could be the most active components compared to others. Further energy analyses of individual components across all simulation systems involving M^pro^ complexed with multiple Rutan compounds highlight the substantial contributions of Coulombic, lipophilic, and Solv_SA energy terms for complex formation. Key residues, including C145, E166, and Q189 of M^pro^ played essential roles in complex formations with the Rutan components. These findings enhance the fundamental understanding of the mechanistic action of polyphenols with antiviral efficacy against COVID-19, paving the way for further exploration of their therapeutic potential.

## Data Availability

The raw data supporting the conclusions of this article will be made available by the authors, without undue reservation.
