# Supplementary material for: Computational and in vitro evaluation of sumac-derived ©Rutan compounds towards Sars-CoV-2 Mpro inhibition
Source: Front Pharmacol. 2025 Feb 4;16:1518463. doi: 10.3389/fphar.2025.1518463 (PMC11832515; doi:10.3389/fphar.2025.1518463)
Supplement: Supplementary file 1 [file Table1.docx]

**Supplementary materials**

**Computational and In Vitro Evaluation of Sumac-Derived ©Rutan Compounds Towards Sars-CoV-2 Mpro Inhibition**

**Muzaffar Kayumov^1,*,†^, Parthiban Marimuthu^2,3,†^, Jamoliddin Razzokov^4,5,6^, Nurkhodja Mukhamedov^1,7^, Akmal Asrorov^1^, Nodir S. Berdiev^1^, Jamolitdin F. Ziyavitdinov^1^, Ansor Yashinov^1,8^, Yuliya Oshchepkova^1^, Shavkat Salikhov^1^, Sharafitdin Mirzaakhmedov^1^**

^1^Institute of Bioorganic Chemistry, AS of Uzbekistan, 83 M.Ulughbek Street, 100125 Tashkent, Uzbekistan

^2^Pharmaceutical Science Laboratory (PSL-Pharmacy) and Structural Bioinformatics Laboratory (SBL-Biochemistry), Faculty of Science and Engineering, Åbo Akademi University, FI-20520 Turku, Finland

^3^Center for Global Health Research, Saveetha Medical College, Saveetha Institute of Medical and Technical Sciences, Chennai 602 105, India.

^4^Institute of Fundamental and Applied Research, National Research University TIIAME, Kori Niyoziy 39, 100000 Tashkent, Uzbekistan

^5^Department of Natural Sciences, Shakhrisabz State Pedagogical Institute, Shakhrisabz Street 10, Kashkadarya 181301, Uzbekistan

^6^Department of Biotechnology, Tashkent State Technical University, Universitet 2, Tashkent 100095, Uzbekistan

^7^Department of Natural Compounds and Applied Chemistry, National University of Uzbekistan, Tashkent 100174, Uzbekistan

^8^Shanghai Institute of Materia Medica, Chinese Academy of Sciences, Shanghai, China

*Authors to whom correspondence should be addressed.

^†^ These authors contributed equally

**Table S1.** The MMGBSA-based energy estimation for five different Rutan compounds interacting with M^pro^

| **Replicates** | **R5** | **R6** | **R7** | **R7`** | **R8** |
| --- | --- | --- | --- | --- | --- |
| 1 | -94.633 | -56.719 | -65.342 | -98.579 | -103.415 |
| 2 | -103.462 | -109.780 | -45.238 | -84.530 | -104.466 |
| 3 | -76.077 | -110.830 | -70.758 | -96.570 | -119.165 |
| 4 | -79.298 | -83.335 | -83.948 | -95.421 | -90.664 |
| 5 | -52.105 | -108.105 | -70.931 | -85.466 | -104.103 |
| **Average** | **-81.115** | **-93.754** | **-67.243** | **-92.113** | **-104.363** |
